# Retraction: Leptin Downregulates Aggrecan through the p38-ADAMST Pathway in Human Nucleus Pulposus Cells

**DOI:** 10.1371/journal.pone.0283028

**Published:** 2023-03-29

**Authors:** The PLOS ONE Editors

The *PLOS ONE* Editors retract this article [1] because it was identified as one of a series of submissions for which we have concerns about peer review, authorship, and similarities across articles. Similarities were also noted between the two GAPDH panels reported in Figure 1B of [1]. These concerns call into question the validity and provenance of the reported results. We regret that the issues were not addressed prior to the article’s publication.

ZL and JS did not agree with the retraction. All other authors either did not respond directly or could not be reached.
